# Post-Ebola Measles Outbreak in Lola, Guinea, January–June 2015^1^

**DOI:** 10.3201/eid2206.151652

**Published:** 2016-06

**Authors:** Jonathan E. Suk, Adela Paez Jimenez, Mamadou Kourouma, Tarik Derrough, Mamadou Baldé, Patric Honomou, Nestor Kolie, Oularé Mamadi, Kaduono Tamba, Kalaya Lamah, Angelo Loua, Thomas Mollet, Molou Lamah, Amara Nana Camara, Vladimir Prikazsky

**Affiliations:** European Centre for Disease Prevention and Control, Solna, Sweden (J.E. Suk, A.P. Jimenez, T. Derrough, T. Mollet, V. Prikazsky);; World Health Organization, Geneva, Switzerland (J.E. Suk, A. Paez Jimenez, T. Derrough, T. Mollet, V. Prikazsky);; World Health Organization, Conakry, Guinea (M. Kourouma, M. Baldé, P. Honomou, N. Kolie, O. Mamadi, K. Tamba, K. Lamah, A. Loua);; Direction Préfectorale de la Santé, Lola, Guinea (M. Lamah, A. Nana Camara)

**Keywords:** Ebola, post-Ebola, hemorrhagic fever, measles, viruses, vaccine, vaccination, immunization, vaccine-preventable diseases, MCV1, Guinea, N’Zérékoré, Lola, Conakry, Africa, Western Africa

## Abstract

During public health crises such as the recent outbreaks of Ebola virus disease in West Africa, breakdowns in public health systems can lead to epidemics of vaccine-preventable diseases. We report here on an outbreak of measles in the prefecture of Lola, Guinea, which started in January 2015.

In 2014 and 2015, Guinea reported 3,804 confirmed and suspected Ebola cases and 2,536 deaths (*1*). The consequences of Ebola include social instability, weakened food security, reduced vaccination coverage, and increased public mistrust of healthcare systems (*2*,*3*).

Lola, a prefecture in Guinea’s forested area, borders Liberia and Côte d’Ivoire and consists of 9 subprefectures and ≈180,000 inhabitants. During September 2014–February 2015, 159 confirmed and probable Ebola cases in Lola were reported; 137 resulted in death. During this time, the World Health Organization (WHO) Regional Strategic Plan for Immunization 2014–2020 routine measles vaccination plan, which aims for 95% coverage by 2017 (*4*), was suspended in areas of Guinea where active Ebola virus transmission was reported (*5*). Furthermore, although supplementary immunization activities are essential to measles vaccination coverage in Guinea, a nationwide catch-up campaign scheduled for the second half of 2014 was interrupted because of the Ebola outbreak and did not reach Lola (*6*). The previous supplementary immunization activities took place in Guinea in 2012, aiming for 95% coverage among children 9–59 months of age (*7*). After that campaign, a cross-sectional survey estimated vaccination coverage to be 61% among this age group in the N’Zérékoré region, which includes Lola prefecture (*7*).

Measles surveillance by the Direction Préfectorale de la Santé (DPS) in Lola is based on standard WHO case definitions (*8*). In January 2015, a total of 8 suspected measles cases were reported in Lola, with no subprefecture exceeding the WHO Regional Office for Africa definition of a suspected outbreak (5 suspected cases in 1 month) (*8*). In February 2015, another 20 suspected measles cases were reported in Lola; 12 were in the subprefecture of N’Zoo. A suspected outbreak was declared, and 6 blood specimens from N’Zoo were sent to the national measles reference laboratory in Conakry. Five samples were confirmed positive for measles (1 sample was inadequate for testing), thereby confirming the measles outbreak according to the WHO Regional Office for Africa definition (>3 laboratory-confirmed measles cases in a health district in 1 month) (*8*). Thereafter, measles transmission intensified, and confirmation of measles cases was symptom-based. In total, DPS reported 702 measles cases in Lola during January 1–June 30, 2015 (Figure). No deaths were reported.

**Figure:**
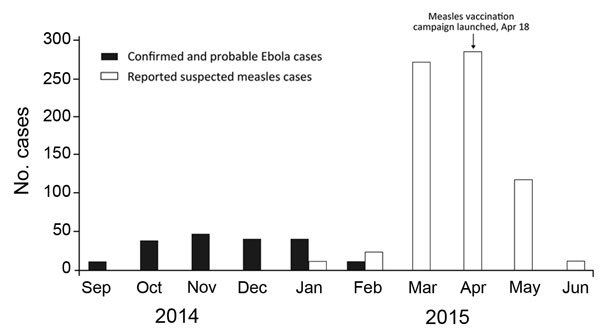
Ebola and measles cases per month in Lola, Guinea, September 2014–June 2015. Ebola data provided by the World Health Organization Global Outbreak Alert and Response Network field team in Lola. Measles data is monthly surveillance data reported by the Direction Préfectorale de la Santé, Lola.

In response to the measles outbreak and in parallel to its Ebola efforts, the WHO Global Outbreak Alert and Response Network (GOARN) field team in Lola worked with DPS to strengthen epidemiologic surveillance, conduct field investigations, assist in social mobilization, and collect case information. Beginning in March 2015, active measles surveillance was reinforced through daily phone calls to regional health centers in each subprefecture of Lola to quickly identify new areas of transmission. In addition, the nongovernmental agency Alima offered free vitamin A supplements to measles patients (*9*), a valuable strategy for areas with poor nutritional levels and low herd immunity. Meanwhile, Guinea received a large shipment of vaccines by the end of March (*6*), but these did not arrive in Lola until mid-April. A vaccination campaign was launched on April 18, with a goal of 95% 1-dose coverage for all children in Lola 6 months to 109 years of age (53,720 children).

The measles case database constructed by the WHO GOARN field team covers every subprefecture in Lola and records cases identified during the early phase of the outbreak, during January 23, 2015–April 4, 2015. Although the case data are not exhaustive (n = 284), the database can be used to analyze transmission dynamics. The average and median age of case-patients was 2.8 years and 2.0 years, respectively, substantially lower than the mean age of infection of 5.6 years in Africa (*10*). Of 284 case-patients, 263 (92.6%) were 0–5 years of age, 17 (6.0%) were 6–15 years of age, and 4 (1.4%) were >15 years of age. This low age distribution is indicative of a disruption in vaccination and also suggests that higher immunity existed among older age groups (*11*), related to either strong pre-Ebola vaccination coverage or prior exposure to measles. Of 281 cases with reported vaccination status, 267 (95%) were not vaccinated and 14 (5%) were vaccinated; health records did not differentiate between 1- and 2-dose coverage.

Persons in the subprefecture of N’Zoo in Lola benefited from active measles surveillance implemented by a well-organized local health team. N’Zoo was also the first subprefecture population in Lola to receive free vitamin A supplements (beginning March 2015). N’Zoo represents 8.7% of the population of Lola (15,559 inhabitants) but accounted for 30% of reported measles cases in Lola (212/702). Because of its efficiency of surveillance, N’Zoo may offer a better estimate of what occurred throughout the prefecture. Extrapolating the incidence rate from N’Zoo across all of Lola yields 2,450 cases. This figure should be treated with caution, however, because measles transmission dynamics cannot be assumed to have been consistent across Lola. Additionally, over-reporting during measles outbreaks, particularly in areas where active surveillance is implemented, is a possibility when cases are diagnosed principally on the basis of symptoms (*12*).

## Discussion

Many barriers to an effective response to measles existed in Lola. Healthcare-seeking behaviors declined markedly during the Ebola epidemic (*2*), which likely contributed to probable underreporting of measles cases, a fact further substantiated by the lack of fatalities reported to DPS. Although there are wide variances in estimates of measles case-fatality rates, a large study has suggested an average case-fatality rate of 3.7% for Africa (*13*), which would correspond with 26 deaths from the 702 suspected cases reported in Lola during January–June 2015. The lack of reported fatalities may also be related to the effect of the Ebola epidemic on burial practices; any families notifying authorities about deaths would be required to conduct “safe and dignified burials,” a protocol that had been met with resistance by many local groups (*2*).

Another barrier to response was the late initiation of the vaccination campaign. When the full shipment of vaccines arrived in Lola in mid-April, logistical planning was challenged by shortages of personnel, fuel for automobiles, and appropriate vehicles for traversing difficult terrain during the onset of the rainy season. The campaign is estimated to have reached 92% of the target population, but persons in some urban areas and villages were reluctant to receive vaccinations. The launch of the campaign coincided with reduced measles transmission (Figure), but further modeling research would be required to assess its effect on the course of the outbreak.

In the aftermath of the Ebola epidemic, discussions about strengthening global outbreak response capacities are ongoing (*14*,*15*). The front lines of disease surveillance and outbreak detection often occur in rural settings that are understaffed and underresourced. As Guinea transitions to a post-Ebola phase, the field presence of public health doctors may be reduced. This reduction would be unfortunate, because the technical and cultural expertise of the doctors from Guinea in Lola and other similar settings transcends Ebola and could be harnessed to support a wide range of public health activities.

Strengthened investments in local public health systems will be essential to ensure the population of Guinea can recover from the Ebola epidemic and be better protected from future disease outbreaks. Aside from personnel, the public health infrastructure, including surveillance, information and communications technology, and temperature-controlled supply chains, particularly requires attention. Meanwhile, great efforts will also be needed to restore and enhance community trust in medicine and public health (*2*).
